# Interleukin-10-alveolar macrophage cell membrane-coated nanoparticles alleviate airway inflammation and regulate Th17/regulatory T cell balance in a mouse model

**DOI:** 10.3389/fimmu.2023.1186393

**Published:** 2023-05-19

**Authors:** Jun-Da Li, Jia Yin

**Affiliations:** ^1^ Department of Allergy, Peking Union Medical College Hospital, Chinese Academy of Medical Sciences, Peking Union Medical College, Beijing, China; ^2^ Department of Allergy, Peking Union Medical College Hospital, Beijing Key Laboratory of Precision Medicine for Diagnosis and Treatment on Allergic Diseases, Beijing, China; ^3^ Department of Allergy, Peking Union Medical College Hospital, National Clinical Research Center for Dermatologic and Immunologic Disease, Beijing, China

**Keywords:** allergic airway disease, interleukin-10, cell membrane-coated nanomaterials, delivery system, house dust mite

## Abstract

**Background:**

Allergic airway disease (AAD) is a chronic disease characterized by airway inflammation, bronchoconstriction, and hyperresponsiveness. Although exogenous interleukin-10 (IL-10) alleviates allergic inflammation, it has a short half-life *in vivo*. Cell membrane-coated nanomaterials have been shown to protect therapeutic payloads and increase therapeutic efficacy.

**Objective:**

This study was aimed at investigating the efficacy of a novel macrophage-based nanoparticle drug for the treatment of house dust mite (HDM)-induced allergic airway diseases.

**Methods:**

IL-10-poly (lactic-co-glycolic acid (PLGA) nanoparticles were encapsulated in alveolar macrophage cell membranes. An allergic airway disease mouse model was established by repeated inhalation of HDM extracts. The mice were treated with free IL-10, IL-10-PLGA nanoparticles (IL10-NP), or IL-10-alveolar macrophage cell membrane-coated nanoparticles (IL10-AMNP). The therapeutic effects were evaluated by measuring airway hyperresponsiveness, lung inflammation, cytokine levels, and regulatory T cells (Treg)- T-helper 17 (Th17) cell balance.

**Results:**

Compared to free IL-10, IL10-AMNP significantly reduced airway hyperresponsiveness and T-helper 2 (Th2)/Th17 cytokines and inhibited neutrophilia and eosinophilia recruitment into the airways of HDM-induced mouse models. Additionally, the balance between Tregs and Th17 cells was significantly improved in groups treated with IL10-AMNP.

**Conclusion:**

This study demonstrated that PLGA nanoparticle cores coated with alveolar macrophage cell membranes can effectively deliver therapeutic cytokines to the lungs and improve the homeostatic balance between Tregs and Th17 cells. These findings suggest that macrophage-based nanoparticle drugs represent a promising approach for treating allergic airway diseases.

## 1 Introduction

Allergic airway diseases, such as hay fever and allergic asthma, are chronic inflammatory diseases characterized by wheezing, coughing, and breathlessness; they affect a significant proportion of the global population and pose a major health burden (1). These diseases are characterized by eosinophilic airway inflammation, mucus hypersecretion, airway hyperresponsiveness, and airway obstruction, which lead to significant morbidity and mortality. Current therapeutic strategies for asthma, such as corticosteroids, have limitations in terms of efficacy and side effects. Recently, several new biological therapies have been developed to control asthma (2).

Immune dysregulation and Th17- Treg cell imbalance have been implicated in the pathogenesis of allergic airway diseases (3, 4). A previous study indicated that the homeostatic balance between Tregs and Th17 cells was markedly altered during asthma exacerbation and correlated with asthma severity (4). Treg cells produce anti-inflammatory cytokines, such as interleukin 10 (IL-10) and transforming growth factor-beta (TGF-β), which suppress inflammation and maintain immune homeostasis (5). IL-10 is a potent anti-inflammatory cytokine that plays a crucial role in maintaining immune homeostasis (6). It inhibits Th17 and Th2 cells and promotes Treg cell differentiation and survival (7, 8). However, clinical studies have shown that IL-10 has a short half-life *in vivo* and presents a mean terminal-phase half-life ranging from 2.7 to 4.5 h (9, 10). Therefore, an effective IL-10 delivery system must be developed that can protect cytokines from degradation and improve their stability.

Nanoscale platforms have emerged as promising tools for the diagnosis and treatment of various diseases (11). PLGA is a commonly utilized biodegradable polymer that has been approved for food and drug applications (12), and it has also been approved as a drug delivery platform in humans owing to its favorable properties, such as good bioavailability, controlled release, and excellent safety profile (13). Cell membrane coating technology is a novel approach that uses natural cell membranes to coat nanoparticle cores, thereby enabling the nanoparticles to evade immune recognition and enhancing targeted delivery to specific cells or tissues (14). Macrophages play a key role in immune surveillance (15). Previous studies have shown that macrophages may play a key role in asthma (2). Macrophages have been suggested as potential indicators of oxidative stress, tissue remodeling, and disease severity in asthma.

In this study, we fabricated and characterized IL-10-alveolar macrophage cell membrane-coated PLGA nanoparticles and investigated their therapeutic potential and ability to regulate the balance between Th17 and Treg cells in a house dust mite (HDM)-induced mouse model of allergic airway disease.

## 2 Methods and materials

### 2.1 Experimental animals

Six- to eight-week-old BALB/c mice were purchased from Charles River Laboratories (Beijing, China) and allowed to acclimate for 1 week in individually ventilated cages at the Laboratory Animal Center of Peking Union Medical College Hospital. The experiments were performed in accordance with guidelines approved by the Institutional Animal Care and Use Committee of Peking Union Medical College Hospital. All groups consisted of six female mice. Our protocol and data reporting followed the ARRIVE guidelines.

### 2.2 Alveolar macrophage membrane harvesting

Bronchoalveolar lavage fluid (BALF) was collected based on a previously described protocol (16). AMs were harvested from the BALF and purified as previously described (17). Briefly, the cell pellets were resuspended and then cultured in DMEM (Gibco, New York, USA) supplemented with 10% FBS, 100 U/mL penicillin, 100 μg/mL streptomycin, and 1 mmol/L glutamine at 37°C with 5% CO_2_. The AMs were enriched after 2 h of adherence. After AMs adhered, cells were collected using a cell scraper after 3 min of digestion with trypsin. The purity was >98% as determined by flow cytometry (**Supplementary Figure S1**). The cells were washed twice with PBS (followed by centrifugation at 300 g for 10 min), and the cell suspension was loaded into a homogenizer at 22000 rpm for 1 min. The cell membrane was collected and centrifuged at 6000 × *g* for 15 min to remove large debris for subsequent experiments.

### 2.3 Preparation and characterization of IL10-NP and IL10-AMNP

The IL-10-nanoparticle (IL10-NPs) were prepared using a double-solvent evaporation method. Briefly, 1 ml of 2%(w/v) PLGA-ethyl acetate solution was mixed with 10µg recombinant murine IL-10 (Cat #210-10, Peprotech, USA) dissolved in 100 µl sterile DNase/RNase-free distilled water. The solution was then converted into an emulsion by sonication. Next, a water-in-oil-in-water emulsion was obtained by adding a 2% PVA aqueous solution and evaporating overnight under stirring to remove the organic solvent. Finally, the IL10-NPs were washed in water by centrifugation at 13000 × *g* for 10 min and resuspended in sterile PBS (pH 7.4). For IL-10-alveolar macrophage cell membrane-coated nanoparticle (IL10-AMNPs) preparation, IL10-NPs were mixed with AM membranes, sonicated at 200 W for 2–3 min, and then extruded using 1000, 400, 200, and 100 nm polycarbonate films.

The IL-10 concentration in IL10-NPs/10-AMNPs was measured using a murine IL-10 ELISA kit (Cat #3432-1H-6, Mabtech, Sweden) according to the manufacturer’s instructions. We calculated the encapsulation efficacy and loading capacity based on the ratio of loaded to total IL-10. Briefly, precisely 1mg of lyophilized NPs was weighed and dissolved in ultrapure water, and the supernatant was used for detecting Free IL10. The remaining part was added to dichloromethane and sonicated for detection of loaded IL-10. encapsulation efficacy = loaded IL-10/(Free IL10 + loaded IL-10) *100%;loading capacity = loaded IL-10/1mg. The membrane coating was confirmed by transmission electron microscopy (TEM; JEM-1400, JEOL Ltd., Japan). The size and zeta potential of the NPs were measured using dynamic light scattering (DLS; Zetasizer Pro, Malvern Panalytical). The IL-10 release rate was measured at each time point (0, 1, 4, 8, 24, 48, and 120 h) using an ELISA kit.

### 2.4 HDM asthma model and treatment protocol

Thirty mice were randomly divided into five groups: negative control (NC), positive control (PC), IL10-NP, IL10-AMNP, and Free IL10. High-endotoxin HDM (Greer, USA; lot: 378908, 192250 EU per vial or 4970 EU/mg) was removed using an endotoxin removal standard spin column (Cat#PUR028, AbD Serotec, UK). After through the column, the concentration of endotoxin was 1569 EU/mg, which was quantified using an Endotoxin Quant Kit (Cat #A39553, Thermo Fisher, USA). Mice were anesthetized with isoflurane and sensitized on days 0, 2, and 4 with 25 µg HDM or PBS intranasally. Challenges were performed with 25 µg HDM in 25 µl PBS from days 14 to 18. The treatment groups received 0.1 µg IL-10 simultaneously with the HDM challenge. The NC group received PBS at all time points.

### 2.5 Measurement of airway hyperreactivity

Airway hyperreactivity (AHR) was measured 24 h after the final challenge as previously described (18) using the FinePointe Series RC and software (DSI Buxco, USA). Airway resistance (RI in cmH_2_O.s/ml) and lung compliance (C in ml/H_2_O) were measured by exposure to PBS as a baseline, followed by increasing concentrations of methacholine (Cat #A2251, Sigma, Mo, USA).

### 2.6 Tissue sample processing

Mice were exsanguinated under terminal anesthesia, and blood was centrifuged at 5000 × *g* for 15 min to obtain serum. For BALF, the airways were flushed 3 times with 0.5 ml of PBS, cells were collected and suspended in PBS for flow cytometry, and the liquid was stored at −80°C for cytokine detection.

The left lung was used to prepare the single-cell suspension. After excision, the lungs were incubated in 200 U/ml collagenase D and 25 µg/ml DNase I (Cat #10104159001, Sigma) at 37°C for 45 min, and then erythrocytes were lysed in ammonium chloride buffer (BD Biosciences, USA). The right upper lung lobe was washed with PBS, fixed in 10% neutral-buffered formalin (Solarbio, Beijing, China), and stained with hematoxylin and eosin (H&E).

### 2.7 Flow cytometry and antibodies

Cells in BALF were stained with CD45-FITC (clone 30-F11), CD11b-APC (clone M1/70), CD11c-PE (clone N418), F4/80-PE-CY7 (clone BM8), and Ly6G-BV421 (clone 1A8, BioLegend) in the presence of an Fc blocker (CD16/CD32, BD Biosciences). A total of 2 × 10^6^ single lung cells were stimulated with cell stimulation cocktail and protein transport inhibitor cocktail (Cat#00-4970-03 and #00-4980-93, eBioscience) in IMDM medium supplemented with 10% FCS, 2 mM L-glutamine, 100 U/ml penicillin-streptomycin for 4 h in a 37°C incubator with 5% CO_2_. The cells were incubated with a Zombie NIR™ Fixable Viability Kit (Cat#423105, BioLegend) at room temperature for 15 min and then stained with antibodies to extracellular antigens in the presence of Fc blocker at 4°C for 25 min. Intracellular and nuclear staining was performed with the Foxp3/Transcription buffer set (Cat# 00-5523-00, eBiosciences). Data were acquired using LSRFortessa and FACSDiva software (BD Biosciences) or Attune NxT 3 L-BRV and Attune NxT software (Thermo Fisher Scientific) and analyzed using FlowJo 10.7.2 (TreeStar, Ashland). The following antibodies were used: CD4-FITC (clone GK1.5), TCR-BV421 (clone H57-597), Foxp3-AF647 (clone MF23), IL-10-PE (JES5-16E3, BD Biosciences), IFN-γ-PE-Cy7 (clone XMG1.2), IL-4-BV605 (clone 11B11), IL-17a-BV510 (clone TC11-18H10.1, Biolegend), and IL-13-PE-eFluor 610 (eBiosciences).

### 2.8 Analysis of cytokines in BALF

The BALF were analyzed to determine the cytokine content, and the levels of IL-5, IFN-γ, TNF-α, IL-2, IL-6, IL-4, IL-10, IL-9, IL-17A, IL-17F, IL-22, and IL-13 were measured using a multiplex assay kit (BioLegend, LEGENDplex™ multiplex assays, Cat#741044) according to the manufacturer’s protocol.

### 2.9 House dust mite-specific antibody detection

Blood was collected and serum was acquired by centrifugation at 3000 × *g* for 10 min. HDM slgG1, sIgG2a, sIgG2b, and sIgE levels were measured using ELISA.

### 2.10 Statistical analysis

Data were analyzed using SPSS (version 22.0; IBM, USA) and GraphPad Prism software (version 9.0; San Diego, CA, USA). All results are expressed as the mean ± SEM. The Mann–Whitney *U* test was used to compare different groups, and *P* < 0.5 was considered significant.

## 3 Results

### 3.1 Fabrication and characterization of IL10-NPs and IL10-AMNPs

**Figure 1:**
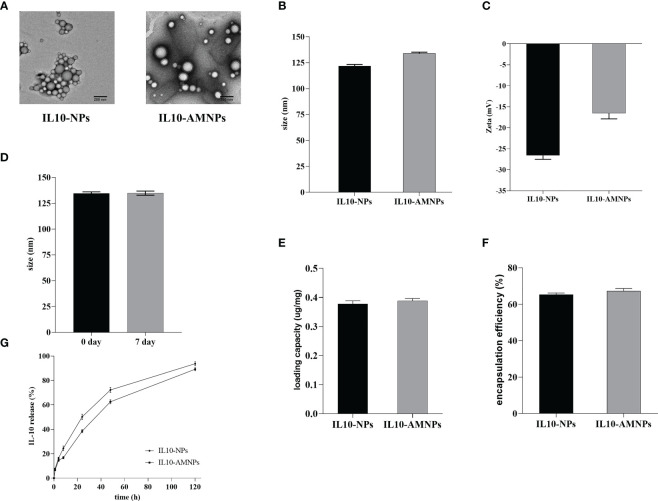
The characterization of IL-10-nanoparticle (IL10-NPs) and IL-10-alveolar macrophage cell membrane-coated nanoparticle (IL10-AMNPs). **(A)** Transmission electron micrographs of IL10-NPs and IL10-AMNPs. **(B)** Size and **(C)** of IL10-NPs and IL10-AMNPs. **(D)** Size of IL10-AMNPs in PBS buffer on day 0 and day. **(E)** Loading capacity and **(F)** encapsulation efficiency of IL-10 in IL10-NPs and IL10-AMNPs. **(G)** *In vitro* cumulative release curve of IL10- NPs and IL10-AMNPs incubated at 37°C in PBS.

In our study, IL10-AMNPs were synthesized by ultrasound using an alveolar macrophage membrane on the surface of PLGA nanoparticles. TEM and DLS were performed to characterize the structure of IL10-NPs and IL10-AMNPs. The TEM results revealed a membrane coating around the PLGA cores and a core-shell structure (**Figure 1A**). In contrast, the IL10-NPs showed no membrane coating. As measured using DLS, the size of the IL10-NP core was ≈120 nm, whereas upon fusion of the AM membranes with the PLGA cores, the diameter of IL10-AMNPs increased from 121.8 ± 1.2 to 133.9 ± 0.9 nm and the surface zeta potential increased from −26.6 ± 0.7 to −16.5 ± 1.1 mV (**Figures 1B, C**). After IL10-AMNPs were suspended in PBS for 7 days, the change in size was not significant (from 133.9 ± 0.9 to 134.9 ± 1.6 nm), indicating nanoparticle stability (**Figure 1D**). The loading capacity of IL-10 was 0.378 ± 0.016 and 0.388 ± 0.012 μg/mg for IL10-AMNPs and IL10-NPs, respectively (**Figure 1E**). The encapsulation efficiency of IL-10 was 65.3 ± 1.3% and 67.3 ± 1.8% for IL10-AMNPs and IL10-NPs, respectively (**Figure 1F**). The IL-10 release profiles were analyzed by incubating at 37°C in PBS, and the IL-10 concentration was measured at seven-time points. IL10-NPs and IL10-AMNPs both showed controlled release capacities for IL-10. IL10-NPs released IL-10 faster than IL10-AMNPs, and the IL-10 release rate reached approximately 90% after 120 h (**Figure 1G**).

### 3.2 IL10-AMNPs suppress airway hyperresponsiveness

**Figure 2:**
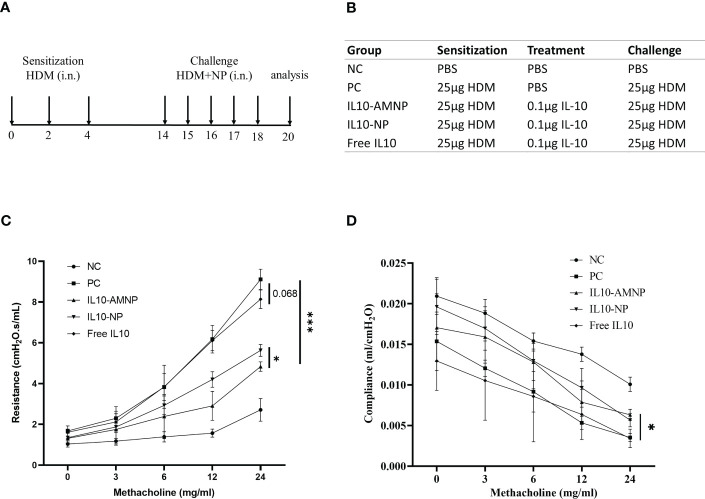
IL10-AMNPs suppress airway hyperresponsiveness. **(A)** Outline of the treatment protocol and **(B)** groups. **(C)** Airway hyperreactivity was measured by FinePointe Series RC system and plotted as airway resistance (RI in cmH_2_O.s/ml) and as **(D)** airway compliance (C in ml/H_2_O) (n = 3). Absolute values are expressed as mean ± SEM (n = 6). *P <.05 and ***P <.001.

We used an HDM-induced mouse model of allergic airway disease to determine the effects of IL10-AMNPs *in vivo* (**Figures 2A, B**). To determine the protective effect of the treatment against the physical manifestations of asthma, we evaluated the effect of IL10-AMNPs on the lung function following HDM exposure. Both the IL10-AMNP and IL10-NP groups showed a tendency toward lower RI levels compared with the positive control and Free IL10 groups. IL10-AMNPs showed greater improvements in airway resistance caused by acetylcholine than the IL10-NP group, although airway resistance in the Free IL10 group was slightly relieved compared to that in the PC group (**Figure 2C**). Furthermore, although a significant reduction in compliance was observed in mice challenged with HDM, the IL10-AMNP and IL10-NP groups showed a slight decrease in compliance and the rates did not differ (**Figure 2D**).

### 3.3 IL10-AMNPs alleviates airway inflammation and inflammatory cell infiltration

**Figure 3:**
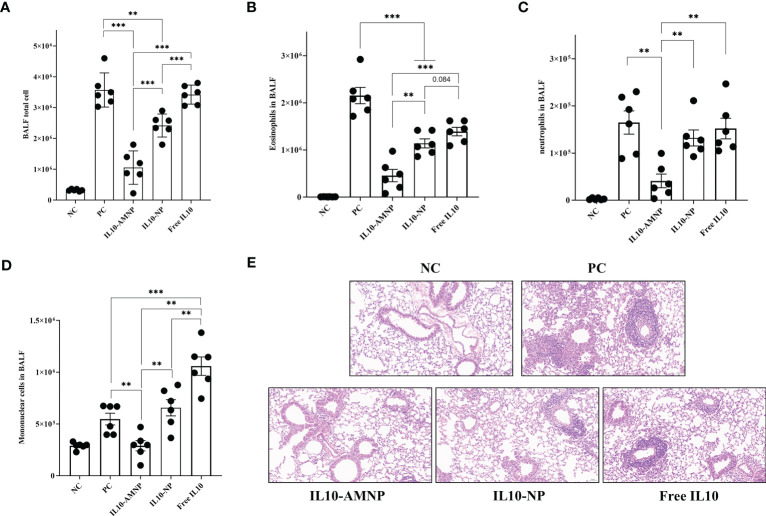
IL10-AMNPs alleviates inflammatory cell infiltration and airway inflammation. **(A)** Total cell, **(B)** eosinophils, **(C)** neutrophils and **(D)** mononuclear cells in BALF. **(E)** H&E staining of the lung tissues in groups. Absolute values are expressed as mean ± SEM (n = 6). **P <.01 and ***P <.001.

To further evaluate the effect of the proposed treatment on pathological changes in the mouse model, BALF cells were analyzed using flow cytometry (**Supplementary Figure S2**) and lung tissues were stained with H&E. HDM drives cell infiltration into the airways and lung tissues, narrows the bronchial trachea, and damages lung structures, including epithelial cells and goblet cell hyperplasia. Significantly more BALF total cells were observed in the PC and Free IL10 groups (**Figure 3A**). While the BALF total cell count was not reduced in the Free IL10 group, eosinophils in the BALF fluid was significantly reduced in the Free IL10 group compared with that in the PC group (**Figure 3B**). After IL-10 nanoparticle treatment, the number of BALF total cells was significantly reduced in the IL10-NP and IL10-AMNP groups. In addition, eosinophils and mononuclear cells in the BALF were reduced in the IL10-NP and IL10-AMNP groups compared with Free IL10 group, although neutrophilia levels were only reduced in the IL10-AMNP group. Surprisingly, the number of mononuclear cells in the BALF were significantly increased in Free IL10 group (**Figures 3B, C, D**). H&E staining showed significantly alleviated narrowing of the bronchial trachea and inflammatory cell infiltration in the IL10-AMNP group compared with that in the IL10-NP and Free IL10 groups (**Figure 3E**).

### 3.4 IL10-AMNPs do not affect HDM-specific immunoglobulin responses

**Figure 4:**
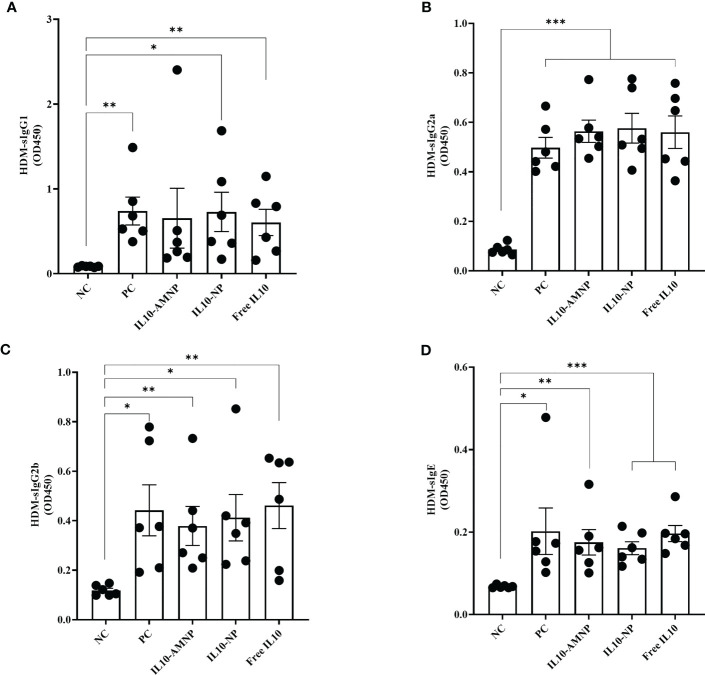
The serum HDM-specific immunoglobulin response after IL-10 drugs treatments. The levels of **(A)** HDM-sIgG1, **(B)** HDM-sIgG2a, **(C)** HDM-sIgG2b, and **(D)** HDM-sIgE were measured by ELISA. Absolute values are expressed as mean ± SEM (n = 6). *P <.05, **P <.01 and ***P <.001.

Next, we determined the effects of IL-10 on HDM-specific immunoglobulins by measuring the levels of HDM-sIgG1, HDM-sIgG2a, HDM-sIgG2b, and HDM-sIgE (**Figure 4**).

We observed a significant increase in the levels of HDM-sIgG1, HDM-sIgG2a, HDM-sIgG2b, and HDM-sIgE in all groups following HDM challenge, although significant changes were not observed among the different treatment groups.

### 3.5 IL10-AMNPs suppress inflammation-related cytokine levels

**Figure 5:**
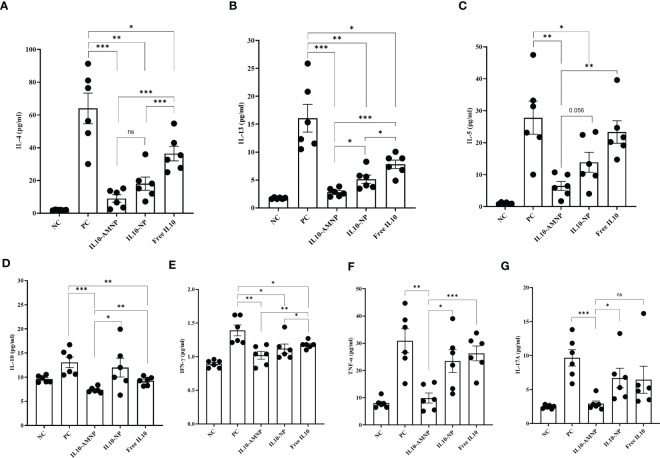
Overview of cytokine profile after IL-10 drugs treatments, measured in BALF. **(A–G)**. Quantification of IL-4, IL-5, IL-13, IL-10, IFN-γ, TNF-α and IL-17A were measured using a multiplex assay kit. Absolute values are expressed as mean ± SEM (n = 6). *P <.05, **P <.01 and ***P <.001.

Exposure to HDM has been shown to elicit a polarized Th2 immune response. Furthermore, repeated inhalation of HDM induces a mixed Th2/Th17 immune response that involves the production of both Th2- and Th17-related cytokines (19). Therefore, we assessed Th1/2/17 cytokine levels in BALF. As expected, the Th2 type cytokines, such as IL-4 and IL-13 were significantly reduced after IL-10 treatment compared with those in the PC group (**Figures 5A, B**). Notably, the IL-13 levels in BALF were lower in the IL10-AMNP group than in the IL10-NP group (**Figure 5B**). After IL-10 nanomaterial application, the IL-5 levels were significantly reduced in the IL10-AMNP and IL10-NP groups compared with that in the PC group (**Figure 5C**). Surprisingly, significant reductions in IL-10 levels were observed in the IL10-AMNP and Free IL10 groups, with lower IL-10 levels in the IL10-AMNP group compared with those in the Free IL10 group (**Figure 5D**). For Th1/17 cytokines, the IFN-γ levels were significantly reduced after IL-10 treatment (**Figure 5E**). Additionally, a significant decrease in TNF-α and IL17A levels was observed in the IL10-AMNP group but not in the IL10-NP and Free IL10 groups (**Figures 5F, G**). Otherwise, the level of other cytokines, including IL-2, IL-6, IL-9, IL17-F, IL-22, were significantly lower than those in PC group (**Supplementary Figure S3**).

### 3.6 IL10-AMNPs decrease Th2 cells and regulate Th17/Treg cell balance

**Figure 6:**
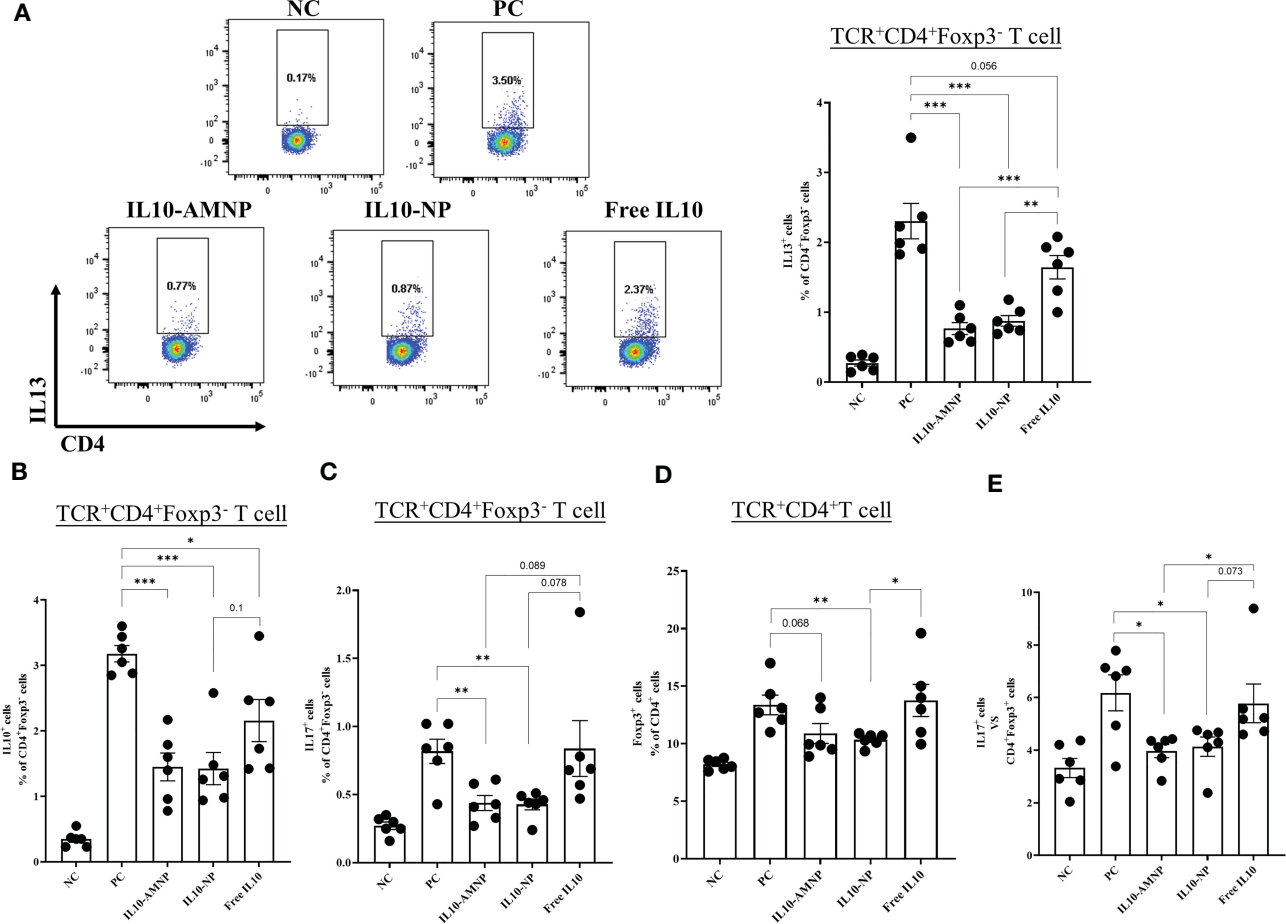
The percentage of Th2 cells and Th17/Treg cell were decreased after IL-10 drugs treatments. The percentages of **(A)** IL13^+^CD4^+^T, **(B)** IL10^+^CD4^+^T, **(C)** IL17A^+^CD4^+^T and **(D)** Foxp3^+^CD4^+^T cells in lung after cell stimulation cocktail and protein transport inhibitor cocktail stimulation. **(E)** the ratio of Th17/Treg cells. Absolute values are expressed as mean ± SEM (n = 6). *P <.05, **P <.01 and ***P <.001.

Th2 and Tregs, which respond to common allergens, play crucial roles in the promotion and suppression of airway inflammation, respectively. Understanding the balance between these cell types is important for understanding allergic airway diseases (20). In our study, a significant decrease in the percentages of IL13^+^CD4^+^T cells was observed in the lungs following treatment with both IL10-AMNPs and IL-10-NPs (**Figure 6A**) and a significant reduction in CD4^+^Foxp3^-^IL-10^+^T cells was observed in both IL-10 treatment groups (**Figure 6B**). Interestingly, the frequency of Foxp3^+^ Treg cells and IL17^+^T cells were significantly increased in the PC group compared with that in NC group (**Figures 6C, D**) whereas the ratio of Th17/Treg was significantly reduced (**Figure 6E**). However, Foxp3^+^ Tregs were decreased in the IL10-NP and IL10-AMNP group compared with that in the PC group, no statistically significant difference was observed between IL10-AMNP and PC group. Meanwhile, the Th17/Treg ratio in the PC group was significantly decreased in the IL10-NP and IL10-AMNP groups (**Figure 6E**).

## 4 Discussion

IL-10 is an important regulatory cytokine that plays a critical role in the control of allergic diseases (6). In previous studies, exogenous IL-10 has been investigated as a potential therapeutic agent for allergic diseases in mouse models (21–23). These studies showed that exogenous IL-10 alleviates allergic inflammation; however, IL-10 has a short half-life *in vivo*. Nanoscale platforms have been extensively studied for the diagnosis and treatment of various diseases (24). These nanomaterials protect drugs from degradation and improve their stability (25). PLGA particles are considered ideal tools for suppressing allergic disease (26). Cell membrane coating technology is inspired by naturally occurring intercellular interactions and has been shown to efficiently guide nanostructures to desired locations, thereby increasing both therapeutic efficacy and safety (27). Macrophage-based delivery systems have shown promise for protecting therapeutic payloads from recognition and clearance by the mononuclear phagocytic system (28). A previous study used macrophage exosome membranes to encapsulate Dnmt3a, and this strategy was successful in treating allergic asthma (29). In this study, we developed a novel approach for treating HDM-induced allergic airway disease in a mouse model by fabricating nanomaterials coated with alveolar macrophage membranes and loaded with an IL-10-PLGA nanoparticle core.

In a previous study, Tournoy et al. (30) demonstrated that endogenous IL-10 suppressed allergen-induced airway inflammation and airway resistance increased in OVA-challenged IL-10^-/-^ mice, although the results were not significantly greater than that in PBS-exposed IL-10^-/-^ mice. Michael et al. (21) found airway resistance was increased in ragweed-induced mice treated with a 25 μg single dose of exogenous IL-10. In our study, the treatment groups were administered 0.1 μg of IL-10 simultaneously with the HDM challenge for a duration of 5 d. Airway resistance was slightly reduced in the Free IL10 group but significantly reduced in the IL10-AMNP group. This finding suggests that IL-10 may require a longer time and a higher dosage to reduce airway resistance compared with IL10-AMNPs.

Airway infiltration by inflammatory cells, particularly eosinophils, is a characteristic feature of allergic airway diseases (31). Claudia et al. (23) found that the intranasal administration of IL-10 inhibited antigen-induced cellular accumulation; however, subcutaneous injections did not have the same effect. This suggests that IL-10 inhibits allergic inflammation and may require a sufficient concentration in the lungs. In this study, IL-10 was administered intranasally. Although the BALF total cell count was not reduced in the Free IL10 group, the number of eosinophils in the BALF fluid was significantly reduced. Compared with a previous study, neutrophils in the BALF were not decreased in the Free IL10 group, which may have been caused by the repeated inhalation of allergens that induced neutrophil infiltration (32). The data show that IL10-AMNPs prevented both eosinophil and neutrophil infiltration into the airways of HDM-challenged mice. This suggests that AM-coated IL-10 nanomaterials may efficiently guide IL-10 into the lungs, improve its half-life *in vivo*, and increase its therapeutic efficacy. Furthermore, our data show the number of mononuclear cells were significantly higher in Free IL10 group. According to a recent study (33), monocyte-derived macrophages can aggregate to injured tissue and be activated through IL-10-dependent mechanism, the short-term, excessive local exogenous IL-10 in the Free IL10 group may lead to increased accumulation of mononuclear cells.

Allergen-specific immunoglobulins, particularly IgE, play important roles in allergic diseases (34). IgE, murine IgG1, and human IgG4 are produced by Th2-type cytokines, and IL-10 can induce a switch toward IgG4 and IgE inhibition, as shown in human studies (35). Elevated IgG4 levels have been associated with controlled allergic diseases in human studies (36). Although studies have found that class switch recombination to IgE occurs in the nasal tissues and bronchial mucosa (37, 38), the class switch always occurs in mature B cells in the germinal centers of the spleen, nodes, and Peyer’s patches (39). In our study, none of the HDM-specific immunoglobulins in serum changed after IL-10 treatment. This suggests that IL-10 and its nanomaterials do not induce an immunoglobulin class switch after intranasal administration.

We successfully established an HDM-induced mixed Th2/Th17 immune response and showed that Th2/Th17 type cytokines in the BALF were significantly increased in the mouse model. The differential effect on the suppression of Th2 type cytokines observed in our study might be the result of the IL-10 PLGA nanoparticle core improving the therapeutic efficacy. Young-Mi et al. demonstrated that dual inhibition of IL17A and TNF-α was effective against neutrophilic inflammation in a mouse model of neutrophilic asthma (40). In the current study, TNF-α and IL17A levels in BALF were only decreased in the IL10-AMNP group, which may explain why neutrophilia infiltration was reduced with IL10-AMNP administration. Surprisingly, IL-10 levels in the BALF were significantly reduced in the Free IL10 and IL10-AMNP groups but not in the IL10-NP group. Considering the sustained release of the IL-10 from the PLGA nanoparticle core, this result may be explained by the fact that excessive local exogenous IL-10 treatment can negatively regulate IL-10 production.

Recent studies have shown that an imbalance in Th17/Treg cells is correlated with asthma severity (41). The deficiency of IL-10 induces Th17 cell differentiation and reduces Treg formation, thus causing allergies, autoimmunity and infection (42). Our data show that the immune response induced by HDM led to an imbalance between Th17 and Treg cells in the lungs. Although the balance between Th17 and Treg cells did not improve in the Free IL10 group, a significant improvement was observed in the IL10-AMNP and IL10-NP groups. This suggests that alveolar macrophage cell membrane-coated IL-10 nanomaterials and IL-10 nanoparticles have the potential to restore the balance between Th17 and Treg cells in the context of HDM-induced allergic airway disease. This is likely due to the improved delivery and targeting of IL-10 to the site of inflammation as well as the increased half-life of IL-10 *in vivo*. Restoration of this balance may also have contributed to the observed reduction in airway resistance and inflammatory cell infiltration. Further research is required to fully understand the underlying mechanisms and potential clinical applications of this approach.

Sex-related differences play a key role in murine models of allergic airway inflammation. Previous studies have demonstrated that in both OVA and HDM mouse models, females exhibit stronger allergic airway inflammation compared to males (43). In this study, we only used female mice for all the indicated experiments. This difference may be mainly due to the differences in sex hormones and innate immune types, which result in a stronger Th2-type inflammation in females, rather than different types of immune responses (44). In our study, female mice, which are known to be more prone to developing allergic airway inflammation, were chosen as the subjects for this research. Considering the potential influence of sex differences on the response to HDM, the only use of female mice is a limitation of this study.

In conclusion, our study demonstrated that the use of IL10-AMNPs effectively improved the therapeutic efficacy of IL-10 against HDM-induced allergic airway disease in a mouse model. IL10-AMNPs significantly reduced airway resistance, alleviated airway inflammation, decreased Th2/Th17 cytokine levels, and improved the Th17-Treg cell balance. These findings suggest that IL10-AMNPs have the potential for use as a therapeutic approach for the treatment of allergic airway diseases. Future studies should focus on optimizing nanomaterial design and dosing strategies to further improve therapeutic outcomes.

## Data availability statement

The original contributions presented in the study are included in the article/**Supplementary Material**. Further inquiries can be directed to the corresponding author.

## Ethics statement

The animal study was reviewed and approved by Institutional Animal Care and Use Committee of Peking Union Medical College Hospital.

## Author contributions

This study was designed by JY. Material preparation, experiment, data collection and analysis were performed by JL. All authors read and approved the final manuscript.
