# Supplementary material for: Interleukin-10-alveolar macrophage cell membrane-coated nanoparticles alleviate airway inflammation and regulate Th17/regulatory T cell balance in a mouse model
Source: Front Immunol. 2023 May 19;14:1186393. doi: 10.3389/fimmu.2023.1186393 (PMC10235466; doi:10.3389/fimmu.2023.1186393)
Supplement: Supplementary file 1 [file DataSheet_1.docx]

Interleukin-10-alveolar macrophage cell membrane-coated nanoparticles alleviate airway inflammation and regulate Th17/regulatory T cell balance in a mouse model

Supplementary Material

## Supplementary Figure


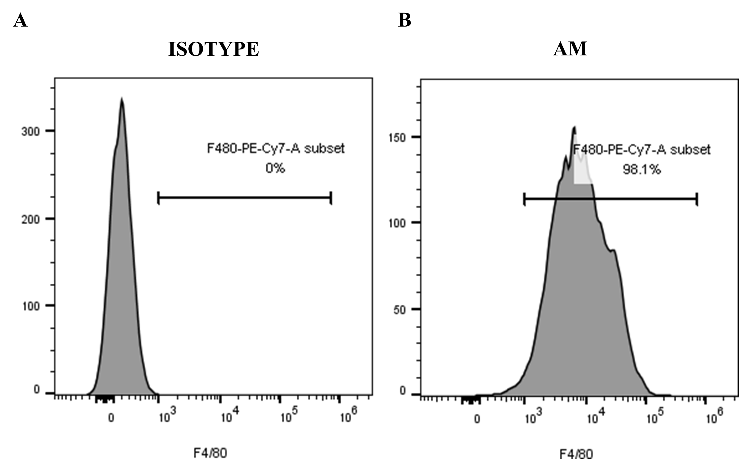


**Supplementary Figure S1.** The purity of Alveolar macrophage cells was assessed using flow cytometry with F4/80 as a marker.


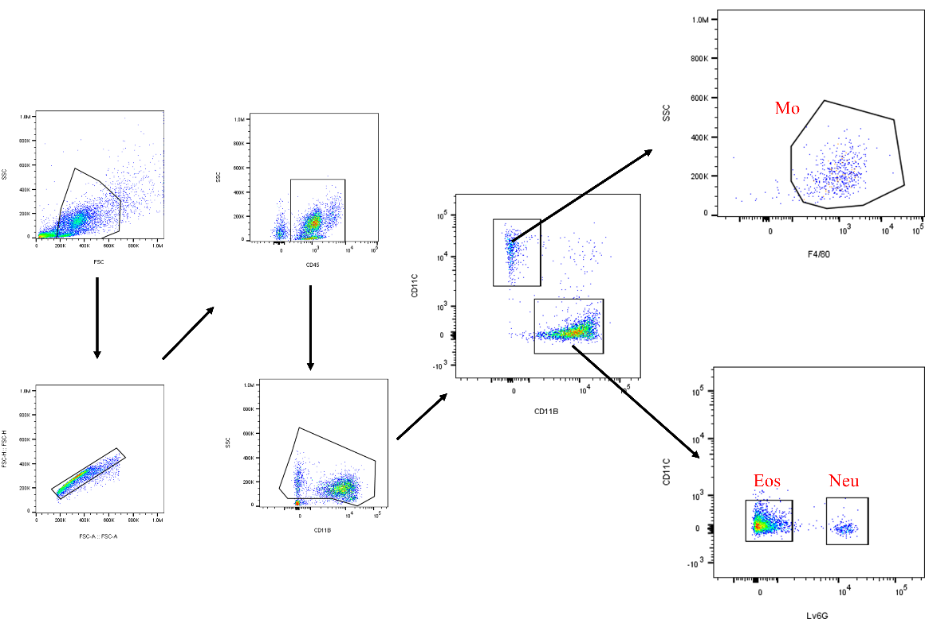


**Supplementary Figure S2.** Gating strategy used to define eosinophils, neutrophils and mononuclear cells in BALF of allergic disease mouse model.  Eosinophils were identified as CD11B^+^CD11C^-^Ly6G^-^ cells. Neutrophils were identified as CD11B^+^CD11C^-^Ly6G^+^ cells. Mononuclear cells were identified as CD11C^+^F4/80^+^ cells.


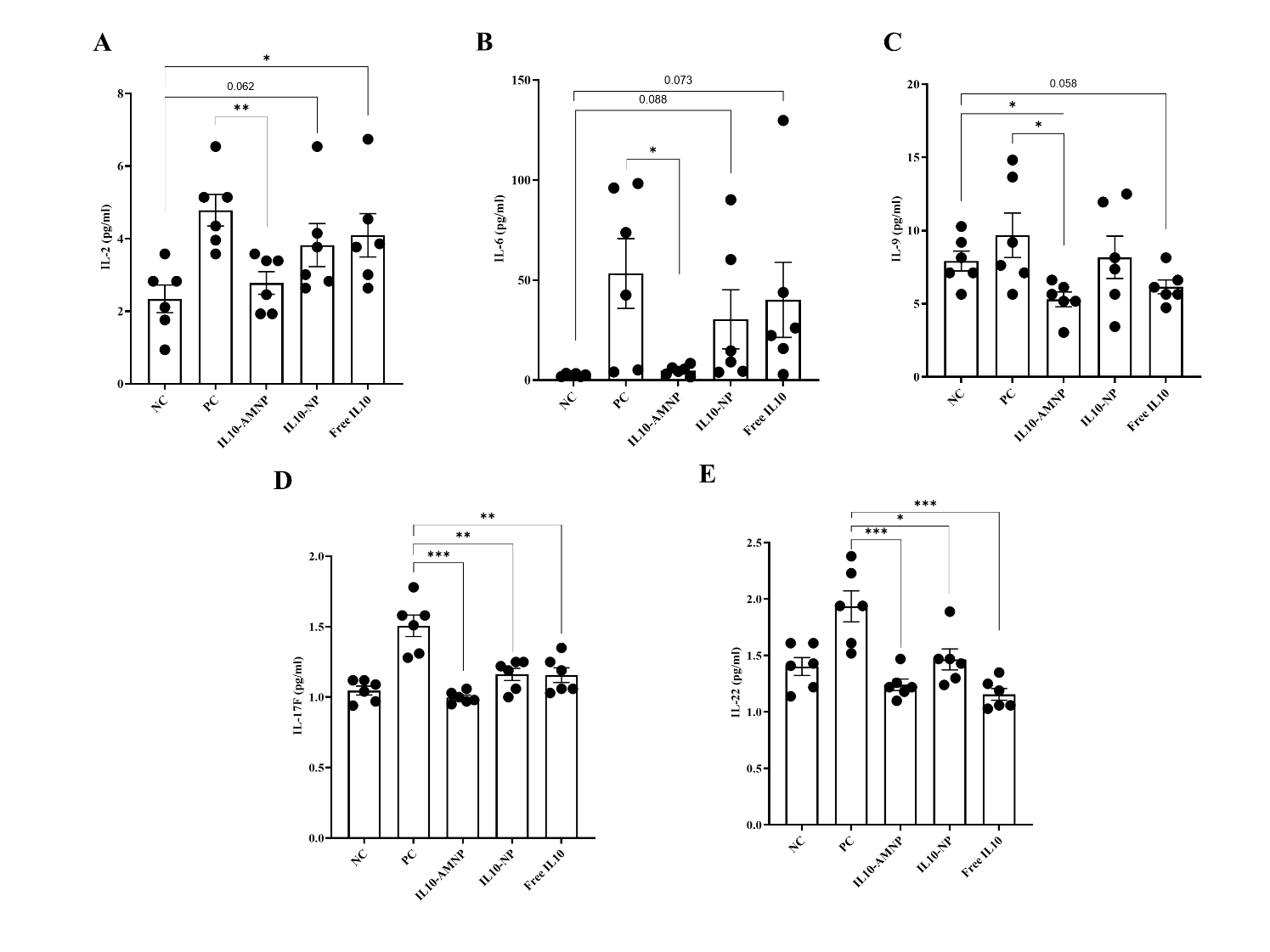


**Supplementary Figure S3.** Overview of cytokine profile after IL-10 drugs treatments, measured in BALF. A-G. Quantification of IL-2, IL-6, IL-9, IL-17Fand IL-22 were measured using a multiplex assay kit. Absolute values are expressed as mean ± SEM (n = 6). *P < .05, **P < .01 and***P < .001.
